# Characteristics of Right Ventricular Blood Flow in Patients With Chronic Thromboembolic Pulmonary Hypertension: An Analysis With 4-Dimensional Flow Cardiovascular Magnetic Resonance Imaging

**DOI:** 10.3389/fcvm.2022.900301

**Published:** 2022-06-15

**Authors:** Wenqing Xu, Xuebiao Sun, Xincao Tao, Dingyi Wang, Yanan Zhen, Xiaopeng Liu, Jing An, Wanmu Xie, Min Liu

**Affiliations:** ^1^Department of Radiology, Peking University China-Japan Friendship School of Clinical Medicine, Beijing, China; ^2^Department of Respiratory and Critical Care Medicine, China-Japan Friendship Hospital, Beijing, China; ^3^China-Japan Friendship Hospital, Institute of Clinical Medical Sciences, Beijing, China; ^4^Department of Cardiovascular Surgery, China-Japan Friendship Hospital, Beijing, China; ^5^Siemens Shenzhen Magnetic Resonance Ltd., Shanghai, China; ^6^Department of Radiology, China-Japan Friendship Hospital, Beijing, China

**Keywords:** cardiovascular magnetic resonance imaging, chronic thromboembolic pulmonary hypertension, 4-dimensional flow, right ventricular function, hemodynamics

## Abstract

**Background:**

Blood flow is closely related to function, but currently, the relationship of right ventricular (RV) blood flow components with RV function and hemodynamics in patients with chronic thromboembolic pulmonary hypertension (CTEPH) remains unclear. Our objective is to qualify RV function with 4-dimensional flow cardiovascular magnetic resonance (4D-Flow CMR) imaging and to investigate the correlation between RV flow and hemodynamics in patients with CTEPH.

**Methods:**

Retrospective enrollment included 67 patients with CTEPH (mean age 47.8±14.2 years, 47 men) who underwent CMR and right heart catheterization (RHC) within 2 days. RHC was used to evaluate hemodynamics. RV flow components including the percentages of direct flow (PDF), retained inflow (PRI), delayed ejection flow (PDEF), and residual volume (PRVo) were quantified on 4D-Flow sequence. RV functional metrics were determined with the CINE balanced steady-state free precession sequence. The sum of PDF and PDEF was compared with RV eject fraction (RVEF). The correlation among RV flow components, RV functional metrics and hemodynamics was analyzed with spearman correlation analysis.

**Results:**

The median (interquartile range) of RVEF, PDF, PDEF, PRI, and PRVo, respectively was 35.5% (18.2, 45.6%), 18% (8.4, 21.4%), 15.1% (13.5, 19.0%), 15.9% (13.8, 20.8%), and 50.6% (35.6, 60.4%). The sum of PDF and PDEF is 35.1% (24.8, 46.6%), which was similar to RVEF (*z* = 0.58, *p* = 0.561). PDF negatively correlated with right ventricular end-systolic volume index (RVESVI), right ventricular myocardial mass index (RVMI) and right ventricular global longitudinal strain (*r* = −0.61, −0.65, −0.64, *p* < 0.001). PRVo positively correlated with RVESVI and RVMI (*r* = 0.50, 0.58, *p* < 0.001). PDF negatively correlated with pulmonary vascular resistance (PVR) (*r* = −0.72, *p* < 0.001) while it positively correlated with cardiac output (CO) and cardiac index (CI) (*r* = 0.64 & 0.52, *p* < 0.001). PRVo positively correlated with mean pulmonary pressure and PVR (*r* = 0.57&0.54, *p* < 0.001). Total five patients died in the perioperative period. RVEF in the deceased patients was similar to survivors (*z* = −1.163, *p* = 0.092). In comparison with the survivors, RVPDF in the deceased patients significantly reduced (*z* = −2.158, *p* = 0.029) while RVPDEF, RVPRI, and RVPRVo in deceased patients were similar to survivors.

**Conclusion:**

4D-Flow CMR can provide simultaneous quantification of RV function and hemodynamics in the assessment of CTEPH without breath-holding. The reduced PDF and increased PRVo were the main characteristics of RV flow in CTEPH.

## Introduction

Chronic thromboembolic pulmonary hypertension (CTEPH) is a multimorphic, progressive, potentially life-threatening pathophysiological condition from non-resolving thromboembolisms of acute pulmonary embolism and a primary cause leading to right heart failure and death in pulmonary hypertension (PH) (1–3). The evaluation of right heart plays a significant role in treatment, prognostic factors and clinical outcomes for CTEPH patients and is routinely assessed by echocardiography in clinics. However, precise evaluation of RV function by echocardiography still remains challenge because of the retrosternal location and complex crescent-shaped geometry of RV and depends on the skill of echocardiographic physician.

Cardiovascular magnetic resonance (CMR) imaging is considered as the gold standard for quantification of ventricular size and function and is increasingly being used to monitor pulmonary hemodynamics and cardiac function in patients with PH. CMR showed a good correlation with the RHC parameters and had high sensitivity and specificity in identifying the underlying causes of PH (4). Meta-analysis (5–7) suggested the decrease in right ventricular ejection fraction (RVEF) was the strongest prognostic factor among all the right ventricular remodeling parameters and was associated with an increase in the risk of clinical worsening and death. Usually, ventricular size, strain, and function are easily obtained with the time-resolved “CINE” balanced steady-state free precession (bSSFP) imaging due to its high reproducibility. Unfortunately, it's hard for patients with severe dyspnea to complete the numerous breath-holds required using standard bSSFP sequences. Thus, accurate evaluation of RV function with CINE is limited for the patients with severe dyspnea.

Flow in 4-dimensional CMR (4D-Flow CMR) that employs a radially undersampled, time-resolved, 3-dimensional, 3-directionally velocity-encoded imaging scheme provides unprecedented capabilities for comprehensive assessment of blood flow, providing various techniques for visualization of blood flow patterns and thus assisting in understanding blood flow changes and retrospectively performing accurate flow measurements (8–12). Previous research suggests that the assessment of ventricular function in healthy subjects with 4D-flow CMR is feasible (13, 14). Roldán-Alzate et al. (15) quantified RV and LV function, pulmonary artery flow with 4D-Flow sequence in a canine model of acute thromboembolic pulmonary hypertension in free breathing.

The above researches indicated that 4D-Flow CMR could be used to evaluate ventricular function in CTEPH patients in free breath. Moreover, the detailed characteristics of RV flow in patients with CTPEH and its relation with hemodynamics are still unclarified. Therefore, we hypothesized that 4D-Flow CMR could supply RV functional metrics by analysis of RV flow components and RV flow components correlated with hemodynamics as well-serum biomarkers of RV dysfunction. Meanwhile, we wondered the difference of RV flow characteristics between the survivors and the deceased patients in perioperative period.

## Materials and Methods

### Study Population

**Figure 1:**
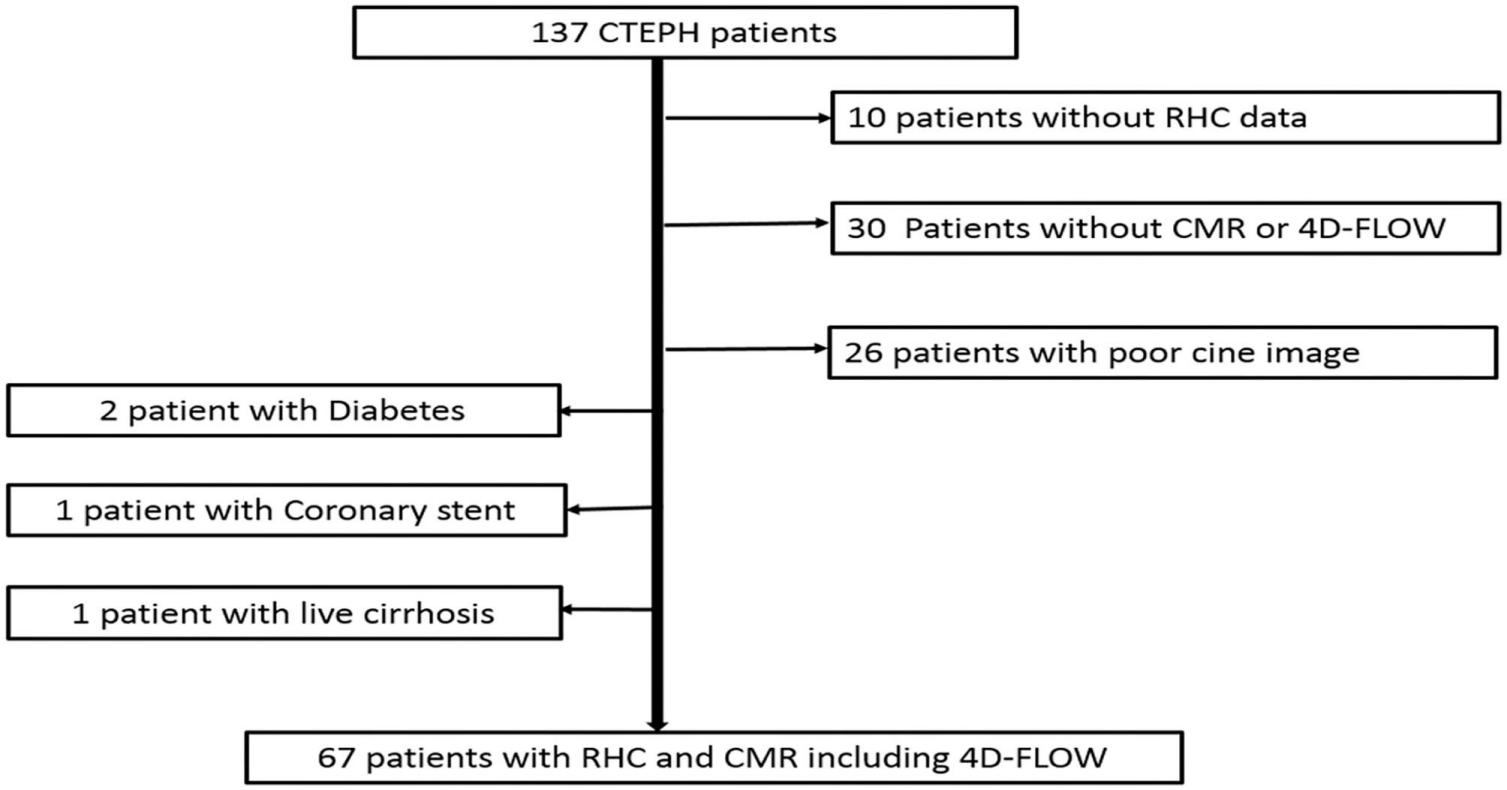
A flowchart detailing how participants were selected.

This study complied with the Declaration of Helsinki and was approved by the ethics committee (IRB No. 2017–24). Informed consent was obtained from all participants or their family. We retrospectively included patients with CTEPH who underwent CMR and right heart catheterization (RHC) in our hospital from January 2018 to January 2021. CTEPH was diagnosed by RHC with pulmonary ventilation perfusion scan and/or computed tomography pulmonary angiography (CTPA). Patients who were older than 18 years and <70 years were included. CMR and RHC were finished within 2 days. CMR included 4D-Flow and CINE sequence. Patients without complete data of RHC or patients who underwent pulmonary thromboendarterectomy (PEA) or Balloon Pulmonary Angioplasty (BPA) before CMR were excluded. Patients with pulmonary artery sarcoma, diabetes, congenital heart disease, and coronary heart disease, malignancy, severe cirrhosis, and kidney dysfunction were excluded. Figure 1 demonstrates a flowchart detailing how participants were selected.

### Cardiovascular Magnetic Resonance Imaging

All patients underwent awake CMR on a 1.5 Tesla clinical scanner (MAGNETOM Area, Siemens Healthcare, Erlangen, Germany) with an 18-channel phased-array surface coil. CMR was acquired during end-expiratory breath holds with retrospective electrocardiographic gating. Standard CINE images in long-axis 4-chamber and contiguous short-axis slices covering both ventricles from base to apex were acquired with True Fast imaging with steady-state free precession sequence in holding-breath (typical acquisition parameters: repetition time/echo time 34.5/1.1 ms, flip angle 50–60°, slice thickness 6 mm, in-plane spatial resolution 1.8 x 1.8mm^2^, temporal resolution 40 ms, 25 reconstructed cardiac phases). And then, 4D-Flow acquisitions were free breathing in sagittal direction, using a 3D retrospectively ECG-triggered, navigator-gated prototype sequence. The field of view was adjusted to cover the whole heart of each subject. Parameters included: repetition time/echo time 5.2/2.5 ms, flip angle 7 degree, reconstructed temporal resolution 40 ms, 2.4 × 2.4 × 2.8 mm^3^ voxel size, and velocity encoding 100–150 cm/s.25 reconstructed cardiac phases were interpolated from 19 to 25 phases based on patients' heart rate ranging from 60 to 80 beats/min. Respiratory navigator gating was used to minimize motion artifacts. The total acquisition time was 6–9 min. Maxwell correction had undergone during scanning. Concomitant gradient field effects were also corrected on the scanner.

### CMR Analysis

Right ventricular functional metrics such as right ventricular end-diastolic and end-systolic volumes index (RVEDVI, RVESVI) and right ventricular ejection fraction (RVEF),. were independently analyzed on CINE images by a radiologist in 4 years of experience with Cardiac function on SyngoVia platform(Siemens Healthcare, Erlangen, Germany).

RV flow components were also independently post-processed and analyzed using CVI^42^ (Version 5.11, Circle Cardiovascular Imaging Inc, Calgary, Alberta, Canada) by a radiologist in 5 years of experience. The analysis of RV flow components consisted of background phase correction, noise filter and velocity aliasing correction, endocardial segmentation at end diastole and end systole with pathline generation from each segmented voxel. The positions of Pathlines at end systole were divided into four functional flow components as described previously (13) such as Direct Flow (DF), Delayed Ejection Flow (DEF), Retained Inflow (RI) and Residual Volume (RVo). The percent of each flow component in right ventricle was the ratio of each component volume and RVEDV including the percent of DF (PDF), the percent of DEF (PDEF), the percent of Retained Inflow (PRI) and the percent of Residual Volume (PRVo).

### Right-Heart Catheterization

All patients underwent right-heart catheterization (RHC) awake. A Swan-Ganz standard thermodilution pulmonary artery catheter was placed at the right inferior pulmonary artery. The measured indices were right atrial pressure (RAP), mean pulmonary arterial pressure (MPAP), Pulmonary Capillary Wedge Pressure (PCWP) and pulmonary vascular resistance (PVR). Cardiac Output (CO) and Cardiac Index (CI) were determined using the Fick method.

### Statistical Analysis

Data are expressed as mean ± standard deviation (SD) or median (interquartile range, IQR). RV flow components were compared using non-parametric 2-independent samples Kolmogorov–Sminov Z tests. Correlations between RV flow components and RV functional metrics measured with CINE, hemodynamics with RHC, the serum N-terminal pro-brain natriuretic peptide (NT-proBNP) level and 6-min walking distance (6MWD)were assessed using the Spearman correlation method. The two-sided significance was set and *p* < 0.05 was considered statistically significant difference. Statistics were analyzed using SPSS 22 (Chicago, IL, USA).

## Results

### Baseline Characteristics

The study included 67 CTEPH patients (mean age = 47.8 ± 14.2 years, 47 men). Table 1 shows the baseline demographic and clinical data of patients with CTEPH. Of the 67 patients, 52 underwent PEA, 12 underwent BPA and the other three patients were treated with Riociguat. Of these five patients deceased in perioperative period including three patients died within 1 week after PEA, one patient died within 1 week after BPA and one patient died of sudden massive hemoptysis during hospitalization. Other 62 patients are still being follow-up.

**Table 1:** Baseline demographic and clinical data of patients with CTEPH.

**Clinical data**	**Mean ±SD/n/median (IQR)**
Age (years)	49 (34, 61)
Gender (M/F)	47/20
Body Mass Index (Kg/m^2^)	20.1 ± 3.4
Heart Rate (b/min)	80 ± 14
Systolic BP (mmHg)	117 ± 13
Diastolic BP (mmHg)	78 ± 11
NT-proBNP (pg/ml)	626 (380, 1,500)
CTNI (ng/mL)	0.02 (0, 0.04)
D-dimer (mg/L)	0.38 (0.16, 0.87)
6 MWD (m)	300 (180, 390)
**NYHA**	
I	3
II	5
III	56
IV	3
Systolic PAP (mmHg)	78 (67, 88)
Diastolic PAP (mmHg)	30 (23, 39)
mean PAP (mmHg)	45 (40, 54)
PCWP (mmHg)	9(7, 11)
PVR (dyn.s.cm^−5^)	1,003 (740, 1,371)
CO (L/min)	2.5 (2.2, 3.3)
CI (L/min/m^2^)	1.5 (1.3, 2.0)

*CTEPH, Chronic thromboembolic pulmonary hypertension; NT-proBNP, N-terminal-pro-B-type natriuretic peptide; CTNI, Cardiac troponin I; 6 MWD, 6-min walking distance; BP, blood pressure; NYHA, New York Heart Association classification; PAP, Pulmonary Arterial Pressure; PCWP, Pulmonary Capillary Wedge Pressure; PVR, Pulmonary Vascular Resistance; CO, Cardiac Output; CI, Cardiac Index*.

### Correlation of RV Flow Components With Function and Biomarkers

**Figure 2:**
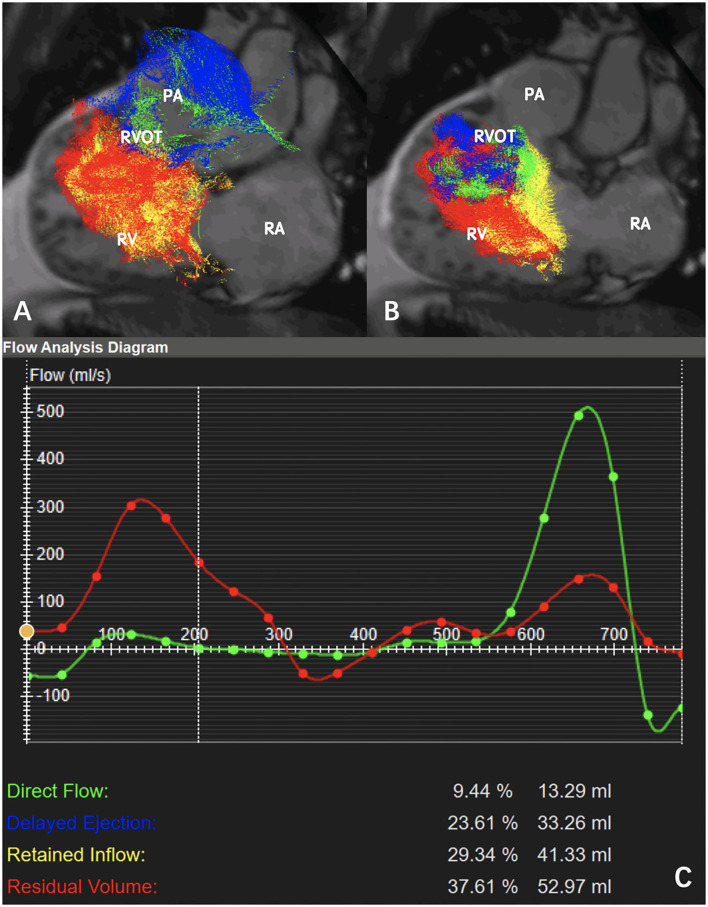
Pathline visualization of right ventricular (RV) flow components in a patient with CTEPH (mean pulmonary arterial pressure = 50 mmHg, pulmonary vascular resistance = 12.26 Wood): **(A)** Pathline visualization of the RV flow components (direct flow, retained inflow, delayed ejection flow, and residual volume) in systole. **(B)** Pathline visualization of the RV flow components (direct flow, retained inflow, delayed ejection flow, and residual volume) in diastole. **(C)** Flow analysis diagram indicated PDF = 9.4%, PRI = 23.6%, PDFE = 29.3%, PRVo = 37.6% (RA: right atrium; RV: right ventricle; RVOT: right ventricular outflow tract; PA: pulmonary artery).

**Figure 3:**
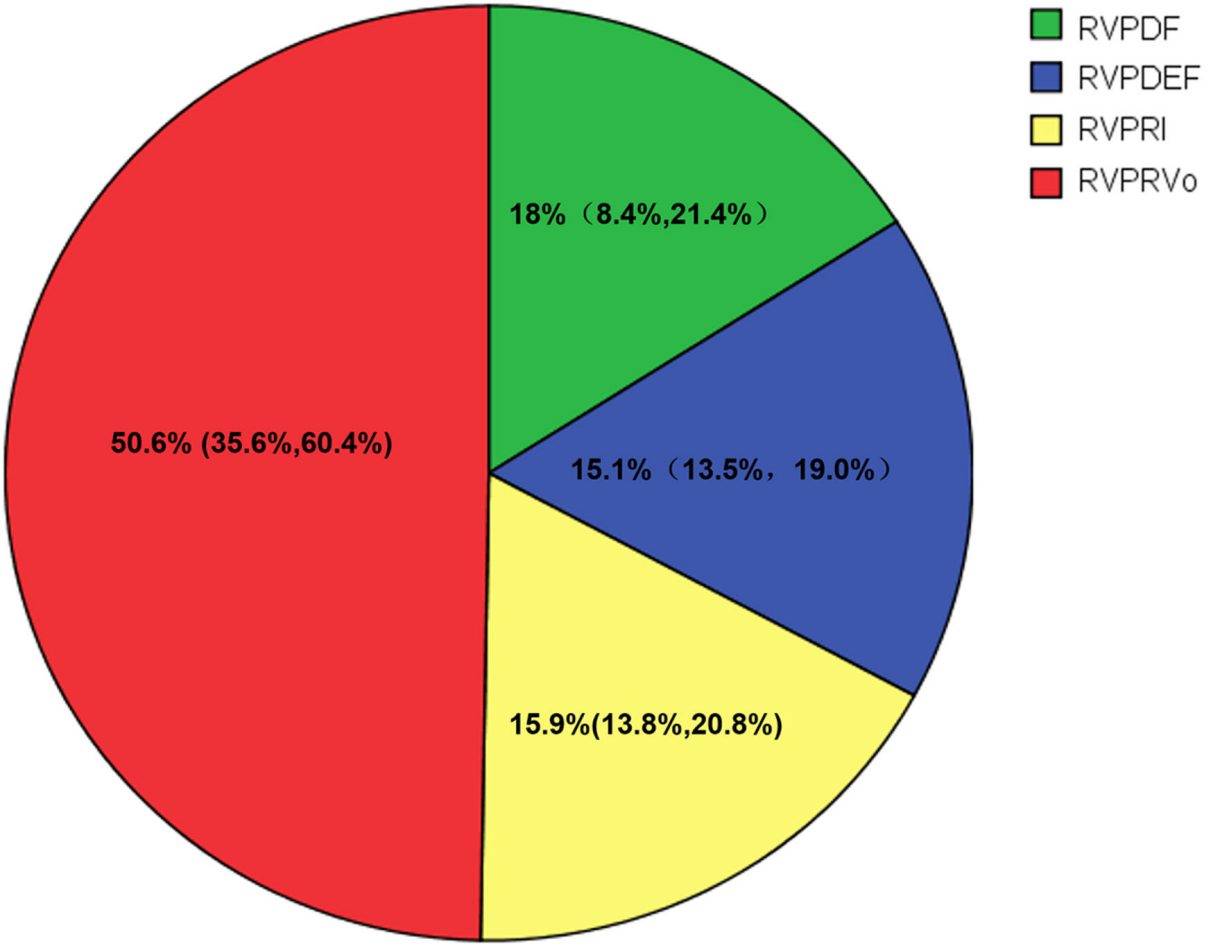
Flow components by percentage of the right ventricular end-diastolic volume (RVEDV) for CTEPH. Green, the percent of direct flow (PDF); Blue, the percent of delayed ejection flow (PDEF); Yellow, the percent of retained inflow (PRI); Red, the percent of residual volume (PRVo).

Visualization of RV flow is shown in Figure 2 and Supplementary Material. Figure 3 shows that the median value of RV flow components such as PDF, PDEF, PRI, and PRVo, respectively was 18% (8.4, 21.4%), 15.1% (13.5, 19.0%), 15.9% (13.8, 20.8%) and 50.6% (35.6, 60.4%). PDF was substantially smaller than PRVo (*z* = 7.82, *p* < 0.001). PRVo was larger than PDEF and RVPRI (*z* = 8.17 and 8.08, *p* < 0.001). PDF (*z* = 1.97, *p* = 0.05), PDEF (*z* = 1.61, *p* = 0.09) was comparable to PRI while PDF was similar with PDEF (*z* = 1.82, *p* = 0.07).

**Figure 4:**
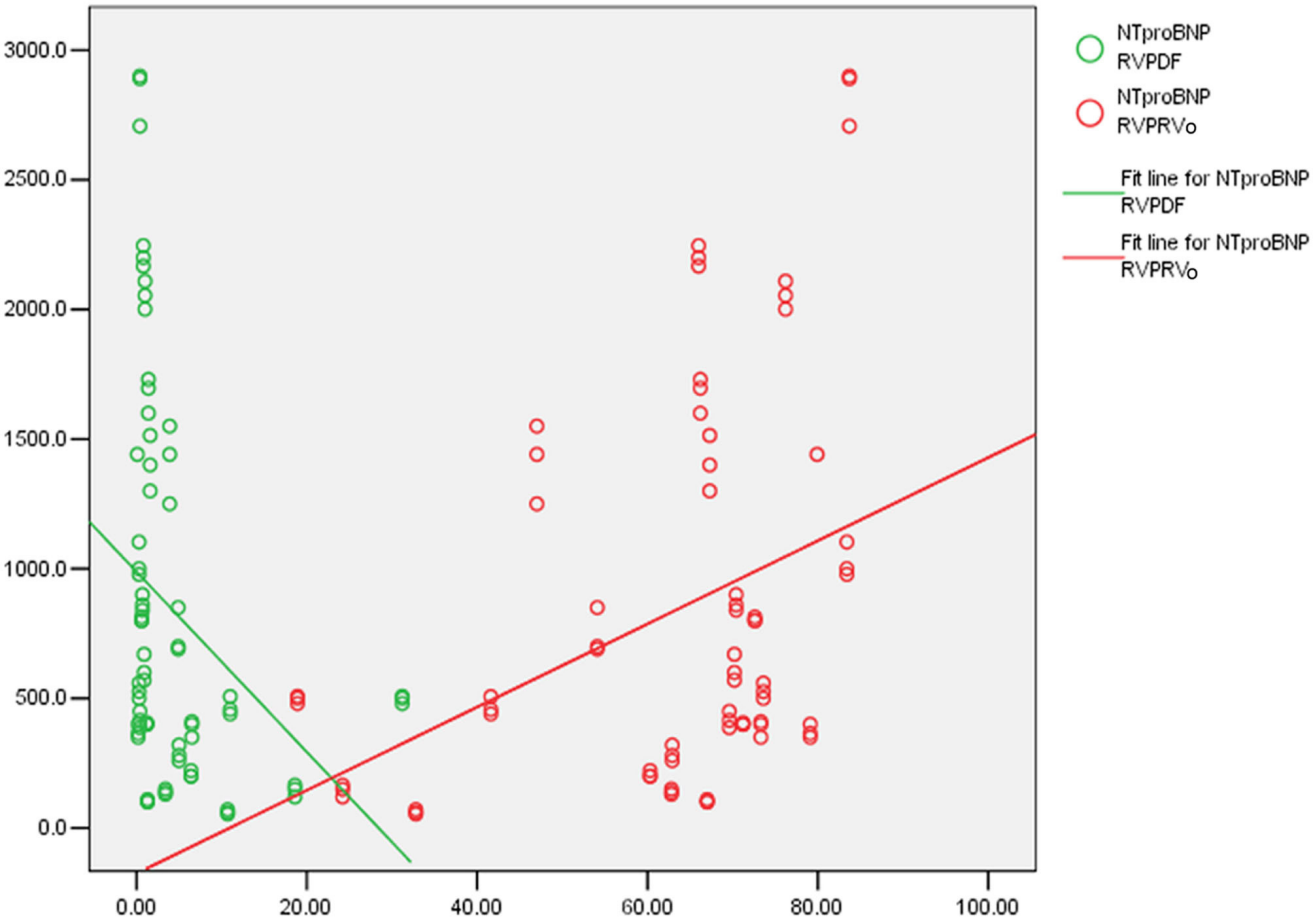
The correlation of the percent of direct flow (PDF), the percent of residual volume (PRVo) and serum N-terminal pro-brain natriuretic peptide (NT-proBNP): (Green) PDF negatively correlated with NT-proBNP. (Red) PRVo positively correlated with NT-proBNP.

Table 2 indicates that PDF negatively correlated with RVESVI, right ventricular mass index (RVMI) and right ventricular global longitudinal strain (RVGLS), positively correlated with RVEF and right ventricular global radial strain (RVGRS) while PRVo positively correlated with RVESVI and RVMI, negatively correlated with RVEF. The sum of PDF and PDEF (PDF+PDEF) is 35.1% (24.8, 46.6%) which is similar to RVEF (*z* = 0.58, *p* = 0.561). Figure 4 shows that RVPDF negatively correlated with the serum N-terminal pro-brain natriuretic peptide (NT-proBNP) *(r* = −0.43, *p* < 0.001) while PRV positively correlated with NT-proBNP (*r*=0.40, *p* = 0.001). Moreover, PDF correlated with 6 MWD (*r* = 0.38, *p* = 0.006) and PRVo negatively correlated with 6 MWD (*r* = 0.41, *p* < 0.001). However, neither PDF nor PRVo correlated with serum D-dimer or CTNI.

**Table 2:** Correlation of right ventricular flow components with functional metrics.

**CMR metrics**	**Median (IQR)**	**PDF (%)**	**PDEF (%)**	**PRI (%)**	**PRVo (%)**
RVEDVI (ml/m^2^)	96.7 (81.5, 124.8)	−0.18 (*p* = 0.120)	−0.25 (*p* = 0.030)	−0.24 (*p* = 0.036)	0.26 (*p* = 0.027)
RVESVI (ml/m^2^)	57.4 (47.0, 83.2)	−0.61 (*p* < 0.001)*	−0.42 (*p* < 0.001)*	−0.42 (*p* < 0.001)*	0.50 (*p* < 0.001)*
RVSVI (ml/m^2^)	32.2 (12.5, 45.0)	0.46 (*p* < 0.001)*	0.26 (*p* = 0.025)	0.26 (*p* = 0.022)	−0.32 (*p* = 0.005)
RVMI (g/m^2^)	23.2 (14.1, 27.3)	−0.65 (*p* < 0.001)*	−0.31 (*p* = 0.008)	−0.32 (*p* = 0.005)	0.58 (*p* < 0.001)*
RVEF (%)	35.5 (18.2, 45.6)	0.71 (*p* < 0.001)*	0.27 (*p* = 0.020)	0.27 (*p* = 0.019)	−0.51 (*p* < 0.001)*
RVGRS (%)	9.7 (6.9, 14.2)	0.50 (*p* < 0.001)*	0.28 (*p* = 0.015)	0.31 (*p* = 0.007)	−0.41 (*p* = 0.002)
RVGCS (%)	−7.2 (−9.1, −5.5)	−0.38 (*p* = 0.002)	−0.26 (*p* = 0.027)	−0.29 (*p* = 0.011)	0.32 (*p* = 0.006)
RVGLS (%)	−11.7 (−16.9, −6.7)	−0.64 (*p* < 0.001)*	−0.16 (*p* = 0.175)	−0.14 (*p* = 0.212)	0.46 (*p* < 0.001)*

^*^Correlation is significant at the 0.001 level (2-tailed). RVEDVI, right ventricular end-diastolic volume index; RVESVI, right ventricular end-systolic volume index; RVSVI, right ventricular stroke volume index; RVEF, right ventricular ejection fraction; RVMI, right ventricular myocardial mass index; RVGRS, right ventricular global radial strain; RVGCS, right ventricular global circumferential strain; RVGLS, right ventricular global longitudinal strain; PDF, the percent of direct flow; PDEF, the percent of delayed ejection flow; PRI, the percent of retained inflow; PRVo, the percent of residual volume.

### Correlation of RV Flow Components With Hemodynamics

Table 3 demonstrates the correlation of RV flow components with hemodynamics measured with RHC. PDF, PDEF, and PRI negatively correlated with PVR while they positively correlated with CO and CI. Moreover, PRVo had a positive correlation with MPAP while it had a negative correlation with CO. None of RV flow components correlated with PCWP.

**Table 3:** Correlation of right ventricular flow components with hemodynamics.

**RV flow components**	**RAP** **(mmHg)**	**MPAP** **(mmHg)**	**PAWP** **(mmHg)**	**PVR** **(dyn.sec.cm-^**5**^)**	**CO** **(L/min)**	**CI** **(L/min/m^**2**^)**
PDF (%)	−0.38 (*p* = 0.002)	−0.47 (*p* < 0.001)*	0.11 (*p* = 0.416)	−0.72 (*p* < 0.001)*	0.64 (*p* < 0.001)*	0.52 (*p* < 0.001)*
PDEF (%)	−0.20 (*p* = 0.117)	−0.32 (*p* = 0.008)	0.11 (*p* = 0.416)	−0.21 (*p* = 0.133)	0.11 (*p* = 0.436)	0.12 (*p* = 0.403)
PRI (%)	−0.18 (*p* = 0.139)	−0.36 (*p* = 0.003)	0.05 (*p* = 0.678)	−0.23 (*p* = 0.098)	0.14 (*p* = 0.291)	0.14 (*p* = 0.331)
PRVo (%)	0.23 (*p* = 0.05)	0.57 (*p* < 0.001)*	−0.01 (*p* = 0.920)	0.54 (*p* < 0.001)*	−0.55 (*p* < 0.001)*	−0.32 (*p* = 0.019)

^*^Correlation is significant at the 0.001 level (2-tailed). PDF, the percent of right ventricular direct flow; PDEF, the percent of right ventricular delayed ejection flow; PRI, the percent of right ventricular retained inflow; PRVo, the percent of right ventricular residual volume; RAP, mean right atrial pressure; MPAP, mean pulmonary arterial pressure; PAWP, Pulmonary Capillary Wedge Pressure; PVR, pulmonary vascular resistance; CO, Cardiac Output; CI, Cardiac Index.

### RV Flow Components and the Perioperative Death

During the perioperative period, five patients died. As shown in Table 4, RVEF in the deceased patients was similar to survivors (*z* = −1.163, *p* = 0.092), while RVEDVI and RVESVI, RVMI in the deceased patients increased significantly. In comparison with the survivors, right ventricular PDF in the deceased patients significantly reduced (*z* = −2.158, *p* = 0.029) while PDEF, PRI, and PRVo in deceased patients were similar to survivors.

**Table 4:** Comparison of right ventricular functional and flow metrics between survivors and deceased patients.

**CMR metrics**	**Survivors (*n* = 62)**	**Dead patients (*n* = 5)**	**z-value**	***p***
RVEDVI (ml/m^2^)	96.0 (81.5, 124.8)	200.1 (102.0, 208.2)	1.664	0.008*
RVESVI (ml/m^2^)	56.1 (47.0, 80.1)	158.6 (61.0, 192.9)	1.659	0.008*
RVSVI (ml/m^2^)	32.2 (13.7, 45.0)	40.1 (17.3, 49.5)	0.995	0.275
RVMI (g/m^2^)	20.2 (13.9, 25.9)	39.7 (30.7, 52.2)	2.277	<0.001*
RVEF (%)	35.5 (18.6, 47.6)	33.7 (27.0, 40.3)	1.163	0.092
RVGRS (%)	11.4 (7.5, 16.3)	6.9 (6.3,9.1)	−1.934	0.053
RVGCS (%)	−8.1 (−9.3, −5.9)	−5.5 (−7.1, −4.2)	−1.683	0.092
RVGLS (%)	−12.2 (−17.2, −6.7)	−6.7 (−8.7, −6.2)	−1.922	0.055
RVSRI	2.0 (1.6, 2.7)	2.4 (1.7, 2.6)	1.221	0.101
RVPDF (%)	18.5 (10.1, 22.0)	14.6 (8.4, 18.0)	−2.185	0.029*
RVPDEF (%)	15.1 (13.5, 18.9)	18.6 (7.3, 28.0)	−1.964	0.054
RVPRI (%)	15.0 (13.8, 20.0)	18.8 (7.9, 29.9)	−1.253	0.211
RVPRVo (%)	51.3 (35.6, 60.6)	48.4 (33.7, 53.2)	−0.991	0.333

* P < 0.05, comparison of CMR metrics between survivors and the dead patients. RVEDVI, right ventricular end-diastolic volume index; RVESVI, right ventricular end-systolic volume index; RVSVI, right ventricular stroke volume index; RVMI, right ventricular myocardial mass index; RVEF, right ventricular ejection fraction; RVGRS, right ventricular global radial strain; RVGCS, right ventricular global circumferential strain; RVGLS, right ventricular global longitudinal strain; RVSRI, Right ventricular end-systolic remodeling index; PDF, the percent of right ventricular direct flow; PDEF, the percent of right ventricular delayed ejection flow; PRI, the percent of right ventricular retained inflow; PRVo, the percent of right ventricular residual volume.

## Discussion

There are four findings in current study: (I) the sum of PDF and PDEF is similar to RVEF measured with CINE sequence. (II) PDF is lower than RVo is the most important RV flow characteristic of CTEPH. (III) PDF and PRVo significantly correlated, but in the opposite direction, with conventional RV functional metrics and hemodynamics. Moreover, PDF significantly reduced in deceased patients in perioperative periods although RVEF was similar between survivors and the deceased patients. To the best of our knowledge, this is the first study to use 4D-Flow CMR to analyze right ventricular flow characteristics in CTEPH.

In previous studies (14, 16–18), the ventricular flow has been categorized into four components. Direct flow (DF) is blood that enters the ventricle during diastole and leaves the ventricle during systole in the analyzed cardiac circle. In healthy subjects, the right ventricular DF represented a larger part of EDV whereas right ventricular residual volume (RVo) was smaller (13). In patients with CTEPH, the decreased PDF and elevated RVo were the most important characteristics of RV flow, meanwhile, PDF was lower than PRVo. This indicated that PDF mainly contributed to the decreased RVEF in patients with CTEPH.

Blood flow is closely related to function. In patients with dilated cardiomyopathy (17), a decrease in left ventricular DF indicated 4D flow-specific markers may detect left ventricular dysfunction even in subtle or subclinical left ventricular remodeling. Moreover, right ventricular PDF also decreased in patients with mild ischemic heart disease (19), indicating that mild impairment of RV function can be detected by 4D flow-specific measures in primary left ventricular disease, but not by the conventional MRI and echocardiographic indices. In patients with CTEPH, with the increase of pulmonary vascular resistance, the decreased right ventricular PDF and increased right ventricular PRVo are the important characteristics of right ventricular blood flow which is similar to alterations of left ventricular flow in patients with DCM (17) or ischemic heart disease (19). This indicates the reduced PDF is not enough for effective systolic ejection by virtue of preserved kinetic energy. Instead of immediate ejection as part of DF, Retained Inflow (RI) resides for at least one cardiac cycle before ejection, and then may be transferred as DEF and RVo. This result is similar to the RV flow characteristics of adult patients with PAH (20) and children patients with PAH (21). Moreover, in children patients with PAH, flow hemodynamic evaluation with 4D-Flow CMR might provide more quantitative insights into vasoreactivity testing in PAH, indicating that 4D-Flow MRI could supply the same vasoreactivity information for patients with CTEPH.

Although RVEF was the strongest prognostic factor among all the right ventricular remodeling parameters in PH, accurate measurement of RVEF on CINE depends on patient's breath-hold during scanning. However, the repeated breath-holding during CMR scan is a very difficult task for patients with severe dyspnea such as PH. 4D-Flow CMR data are acquired using adaptive diaphragm navigator gating without need of breath-holding. Both DF and DEF were flow components which leaves the ventricle during systole in the analyzed heartbeat. In our research, 26 patients were excluded for poor cine image quality from breath artifact, meanwhile, RVEF measured on cine images has no significant difference with the sum of PDF and PDEF measured with 4D-Flow which are consistent with the previous research in patients with PAH (20). Thus, the analysis of RV flow components with 4D-flow CMR can supply right ventricular functional metrics in free-breath status for patients with CTEPH.

The degree of RV structural remodeling and functional adaptability has been demonstrated to be important determinants of functional capacity and survival in patients with CTEPH. RVESVI, RVMI and strain et al. have been used as RV remodeling markers (22, 23). Our finding is that both RV PDF and PRVo correlated, but in opposite directions, with the conventional remodeling parameters such as RVEF, RVESVI, RVMI, and global longitudinal strain. Circulating blood biomarkers such as B-type natriuretic peptide or NT-proBNP have confirmed the clinical prognostic marker. RV PDF negatively correlated and PRVo positively correlated with NT-proBNP. These findings indicated that the decreased PDF and increased PRVo could serve as markers of RV remodeling. Previous studies indicated that 4D-Flow CMR could provide a non-invasive estimate of PVR or MPAP (12, 24). We found both PDF and PRVo correlated, in inverse direction, with pulmonary arterial pressure and PVR. To our knowledge, our work is the first study to demonstrate the correlation of RV flow components with hemodynamics. Moreover, the previous researches showed that right ventricular end-systolic remodeling index (RVSRI), as one of RV remodeling markers, strongly predicted outcomes in PAH and CTEPH (25, 26). When comparing RV functional and flow metrics between the survivors and the deceased patients, we found that RVEF, PDEF, and PRI in survivors and the deceased patients were comparable. However, PDF in the deceased patients reduced and RVEDVI, RVESVI, RVMI increased in comparison with the survivors. This result indicated, in CTEPH patients with similar RVEF, right ventricular PDF may contribute to the poor short-term prognosis.

Our study showed the benefit of using 4D flow CMR to quantify RVEF for CTEPH patients is that quantification can be performed in free breath, however, there were several limitations. First, this was a single-center study performed at a large tertiary hospital, so the inherent limitations of this study design cannot be avoided. Our study only included CTEPH patients, whether current findings can be extrapolated to other etiologies of PH remains unclear. Second, because RHC can't be routinely performed in healthy population, no healthy subjects were included as normal controls in this research to compare with CTEPH patients. Third, due to the limited cases, the role of RV flow components in assessment of prognosis requires further study, although PDF significantly reduced in the deceased patients in current research.

## Conclusion

The reduced Direct Flow and increased Residual Volume were the important characteristics of RV flow in patients with CTEPH. 4D-Flow CMR can provide simultaneous quantification of RV function and hemodynamic parameters in the assessment of CTEPH without breath-holding. Therefore, we recommend 4D-Flow as a routine technique to evaluate CTEPH.

## Data Availability Statement

The raw data supporting the conclusions of this article will be made available by the authors, without undue reservation.

## Ethics Statement

The studies involving human participants were reviewed and approved by the Ethics Committee of China-Japan Friendship Hospital (IRB No. 2017-24). The patients/participants provided their written informed consent to participate in this study.

## Author Contributions

Conceptualization: ML and WXi. Data curation: WXu, XS, XT, and JA. Formal analysis: WXu and XS. Funding acquisition: ML. Investigation: WXu, XS, XT, DW, YZ, and XL. Methodology: ML, DW, and WXi. Supervision: DW and YZ. Writing—original draft: WXu. Writing—review and editing: ML, WXi, and YZ. All authors contributed to the article and approved the submitted version.

## Funding

This work was supported by Elite Medical Professionals project of China-Japan Friendship Hospital (ZRJY2021-BJ02), Medical and Health Science and Technology Innovation Project of Chinese Academy of Medical Science (2021-I2M-1-049), the National Natural Science Foundation of China (81871328), and the Beijing Nature Science Foundation (7182149).

## Conflict of Interest

JA was employed by Siemens Shenzhen Magnetic Resonance Ltd. The remaining authors declare that the research was conducted in the absence of any commercial or financial relationships that could be construed as a potential conflict of interest.

## Publisher's Note

All claims expressed in this article are solely those of the authors and do not necessarily represent those of their affiliated organizations, or those of the publisher, the editors and the reviewers. Any product that may be evaluated in this article, or claim that may be made by its manufacturer, is not guaranteed or endorsed by the publisher.

## Supplementary Material

Supplementary Material Visualization of right ventricular (RV) flow components in a patient with CTEPH (mean pulmonary arterial pressure = 50 mmHg, pulmonary vascular resistance = 12.26 Wood).
